# Assessment of Optimal Stent Implantation with the Use of Optical Coherence Tomography in Patients with Coronary Artery Disease

**DOI:** 10.3390/diagnostics16050813

**Published:** 2026-03-09

**Authors:** Alexandros Kaperonis, Alexandru Scafa-Udriște, Cosmin Mihai, Vlad Bataila, Bogdan Marian Drăgoescu, Vlad Ploscaru, Diana Zamfir, Radu Popescu, Daniel Tonu, Lucian Calmac

**Affiliations:** 1 Department of Cardiology, University of Medicine and Pharmacy Carol Davila, 8, Eroii Sanitari, 050474 Bucharest, Romania; alexandroskap@gmail.com (A.K.); marian-bogdan.dragoescu@drd.umfcd.ro (B.M.D.); diana_zam74@yahoo.com (D.Z.); radu.popescu300397@gmail.com (R.P.); 2 Department of Internal Medicine, General Hospital of Eastern Achaia—Unit of Aigio, Ano Voulomeno, 25100 Aigio, Greece; 3 Emergency Clinical Hospital, 8, Calea Floreasca, 014461 Bucharest, Romania; cosmihalp@yahoo.com (C.M.); vladbataila@yahoo.co.uk (V.B.); vlad_ploscaru86@yahoo.com (V.P.); danieltonu1994@gmail.com (D.T.); lucian.calmac@drd.umfcd.ro (L.C.)

**Keywords:** optical coherence tomography (OCT), intravascular imaging, coronary artery disease (CAD), percutaneous coronary intervention, coronary artery disease

## Abstract

**Background/Objective**: Percutaneous coronary intervention (PCI) has a pivotal role in the treatment of coronary artery disease (CAD). Although PCI is generally guided only angiographically, advancements in intravascular imaging, particularly in optical coherence tomography (OCT), may offer significant advantages. OCT provides high-resolution cross-sectional images that allow for a more detailed assessment of lesion characteristics and procedural outcomes, which are not fully available with angiography. These findings are associated with or predictive of major adverse cardiovascular events (MACE), encouraging the use of OCT in PCI procedures. This study sought to characterize the role of post-PCI OCT imaging in PCI optimization in patients with CAD. **Methods**: This retrospective study includes patients who underwent OCT-guided PCI. A total of 64 patients with various types of CAD were included. The primary endpoint was the identification of suboptimal stent implantation as evaluated with OCT after stent implantation, and the secondary endpoint was the assessment of the possibility to achieve optimal stent implantation after further OCT-guided optimization based on standard definitions of optimal PCI. **Results**: In total, 73 vessels were studied, 42.46% (31) had a stent expansion index (SEI) of < 80%, 31.51% (23) had an SEI between 80–90%, and 26.03% (19) had an SEI of more than 90%. Minimum stent area (MSA) of more than 4.5 mm^2^ was found in 82.19% (60) of vessels, while 17.80% (13) had an MSA below the cut-off value. Suboptimal stent implantation was identified in 35.61% (26) of vessels, including underexpansion 9.58% (7), malapposition 15.06% (11), stent edge dissection 6.85% (5), plaque burden or lipid-rich pool in the stent edges 2.73% (2), and tissue protrusion 1.36% (1). Post-PCI OCT optimization resulted in significant improvements, with only 6.84% (5) of the vessels still not achieving all OCT criteria for optimal stent implantation. **Conclusions**: In patients with CAD, post-PCI OCT evaluation provided useful information, otherwise unavailable by angiography alone. We identified that 35.61% (26) of the targeted vessels, were suboptimally stented. OCT imaging was able to provide procedural and strategic guidance for optimization until the appropriate results, based on our criteria, were achieved in most of the lesions.

## 1. Introduction

Percutaneous coronary intervention (PCI) constitutes an important step in the management of coronary artery disease (CAD). Although angiography-guided PCI is more frequently used, progress in endovascular imaging technology has offered potential advantages. Optical coherence tomography (OCT) provides high-resolution, cross-sectional images, enhancing the evaluation of lesion characteristics as well as the procedural efficacy [1]. OCT provides information not available with angiography alone, such as anatomical lumen characteristics, plaque morphology, and procedural attributes, including stent expansion and apposition, vessel dissections, or adequate lesion coverage [2,3]. These findings have been proven to be associated with or predictive of major adverse cardiovascular events (MACE), validating the use of OCT imaging as an important tool during PCI [4].

Despite the growing body of evidence about the value of intravascular imaging, its routine implementation in everyday practice remains as low as 15% [5]. More specifically, empirical data assessing the additional benefits of OCT evaluation and subsequent optimization during PCI are still scarce [6]. Modern research is especially needed to measure the frequency and types of OCT-defined suboptimal stent implantation following angiographically satisfactory PCI and assess the possibility of using OCT-derived corrective techniques to achieve optimal results. Thus, by analyzing the frequency and mechanisms of OCT-defined suboptimal stent implantation and by investigating the efficacy of OCT-guided optimization in reaching a consensus on defined criteria for optimal stent deployment, the current study sought to evaluate the value of OCT imaging in enhancing PCI quality, focusing on post-PCI OCT findings and their influence on procedural decision-making.

## 2. Methods

### 2.1. Study Population

In this retrospective analysis, we report all the patients who underwent PCI with OCT guidance at the Clinical Emergency Hospital of Bucharest between 2022 and 2023. Clinical data was obtained from the patient’s medical history, physical examinations, and laboratory testing. According to the atherosclerosis study [7], all types of CAD were included (ST segment elevation myocardial infarction [STEMI], non-ST segment elevation myocardial infarction [NSTEMI], unstable angina [UA], or stable angina). For acute coronary syndrome patients, we evaluated the non-culprit lesions exclusively after appropriate treatment of the culprit lesion during the acute phase. Additionally, we included all coronary vessels except for the left main coronary artery. Angiography was performed on all patients using radial access, and a total of 73 vessels were stented. A retrospective analysis of the available data regarding the baseline characteristics of the patients and vessels, together with intra-procedural information from the OCT imaging of the vessels, was performed.

### 2.2. Optical Coherence Tomography Image Acquisition and Analysis

OCT images were acquired with the ILUMIEN OPTIS OCT Intravascular Imaging System (Abbott—St. Jude Medical, St. Paul, MN, USA), using compatible catheters (C7 Dragonfly and Dragonfly OPTIS, St. Jude Medical, St. Paul, MN, USA). An integrated automated pullback device was used at 20 mm/s to scan the entire length of the vessel of interest. During the pullback, continuous flushing of contrast medium directly from the guiding catheter was applied to displace blood from the imaging site.

All PCI procedures were preceded by OCT imaging, and the results were presented as the OCT baseline vessel characteristics. This information was later used during PCI according to the attending physician’s decision regarding balloon and stent sizing or lesion preparation. Interventions included pre-dilatations, stent implantations, and post-dilatations. After acceptable angiographic results, post-PCI OCT was performed, and additional maneuvers were implemented when necessary, according to the OCT results.

OCT recordings were stored using proprietary software (St. Jude Medical, St. Paul, MN, USA) and analyzed offline retrospectively for this study. Analysis of cross-sections was performed together with automated software, which highlighted the lumen contour and stent struts throughout the stented and reference segments of interest. The software’s automatic lumen contour detection was manually corrected before measurements if needed.

**Figure 1:**
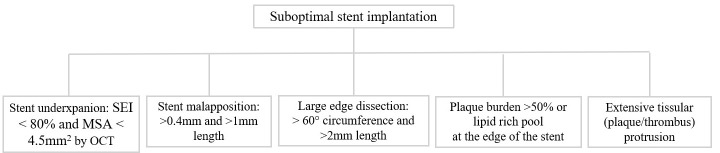
Suboptimal stent implantation criteria.

Qualitative OCT assessment was performed on all stented vessels. Optimal stent implantation was evaluated based on the European Association of Percutaneous Cardiovascular Interventions EAPCI consensus document criteria [8], as summarized in Figure 1. Measurements of proximal and distal lumen reference areas were performed. The minimum stented area (MSA) was then identified by the software, and the stent expansion index (SEI) was calculated using the formula MSA/([proximal reference area + distal reference area]/2). We defined five categories of suboptimal stent implantation. Stent underexpansion was defined if SEI < 80% and MSA < 4.5 mm^2^ [9]. Stent malapposition was considered significant when the distance from the struts to the adjacent lumen was more than 0.4 mm and the longitudinal extension was >1 mm. Edge dissections were considered significant if they exceeded lateral extension > 60°, as well as length > 2 mm. Significant disease at the stent edges was defined as >50% plaque burden or a lipid-rich pool. Moreover, the EAPCI consensus statement recognizes the presence of tissue protrusion (plaque or thrombus), defined as tissue ≥ 500 μm post-PCI through stent strands, as a marker of poor prognosis in the case of elective or urgent PCI. Finally, we evaluated the presence and severity of calcified lesions using the following OCT criteria: presence of calcium thickness > 0.5 mm, angle > 180° of vessel arc, and length > 5 mm.

### 2.3. Study Workflow and OCT-Guided Stent Optimization Protocol

**Figure 2:**
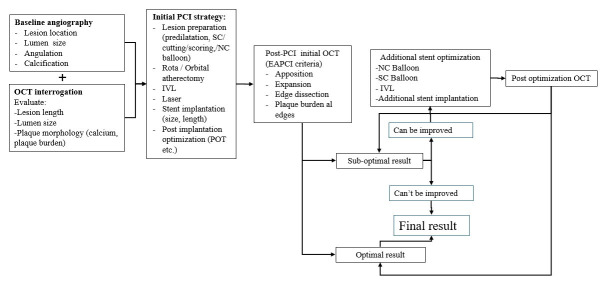
Stepwise OCT-guided PCI workflow. SC balloon, semi-compliant balloon; NC balloon, non-compliant balloon; IVL, intravascular lithotripsy; POT, proximal optimization technique; ROTA, rotablation.

In this study, a stepwise OCT-guided PCI workflow was employed. Baseline angiography was integrated with pre-intervention OCT to characterize lesion anatomy, vessel dimensions, and plaque morphology, thereby guiding lesion preparation and stent selection. Post-PCI OCT was routinely carried out to evaluate stent expansion, apposition, and lesion coverage after angiographically acceptable stent implantation (Figure 2). The above-described predefined suboptimal stent implantation criteria (Section 2.2) were used to interpret the post-PCI OCT results. OCT-guided optimization was carried out whenever a criterion for suboptimal implantation was found. Non-compliant (NC) balloons sized in accordance with OCT-derived reference lumen dimensions were used to treat stent underexpansion with high-pressure post-dilatation. Moreover, NC or semi-compliant (SC) balloons were used to correct stent malapposition. To ensure sufficient lesion coverage, additional stent implantation was performed in cases of significant edge dissection or residual disease at the stent edges. Following optimization, additional OCT imaging was carried out to verify whether the initially detected abnormality was corrected and that the ideal stent implantation was achieved.

### 2.4. Endpoints

The primary endpoint of this study was to identify lesions with OCT-defined suboptimal stent implantation, according to the established criteria, post initial post-PCI OCT evaluation after seemingly angiographically appropriate results. The secondary endpoint was to evaluate the possibility of achieving the OCT criteria for optimal stent implantation after OCT-guided stent optimization.

### 2.5. Statistical Analysis

Statistical analysis was performed using Gretl version 1.9.4 (econometric software for the GNU generation). Data is presented as counts or percentages (%), mean ± standard deviation (SD), or median with interquartile range (IQR), depending on the normality of the data. Normality of the data was evaluated using the Shapiro–Wilk test.

## 3. Results

### 3.1. Patient Baseline Characteristics

A total of 64 patients were included, evaluated and summarized in Table 1. The mean age of patients was 63.03 ± 0.40 years. Moreover, 79.69% were men, 21.87% had diabetes, 84.37% had hypertension, 87.50% had dyslipidemia, and 65.63% were ever smokers. History of angina and previous acute coronary syndrome (ACS) was found in 53.97% and 29.69%, respectively.

Most patients presented with STEMI 53.12%, followed by UA 37.50%, NSTEMI 6.25%, and stable angina 3.13%. Based on angiographic appearance, 26.56% of patients had single vessel disease, 60.94% had double vessel disease, and finally 12.50% had triple vessel disease.

### 3.2. Lesion and Treatment Characteristics

Lesion and procedural characteristics during PCI are summarized in Table 2. We analyzed 73 vessels that underwent stent implantation with OCT guidance. More than half of the lesions, 64.38% (47), were localized in the left anterior descending artery, followed by right coronary artery at 20.55% (15). Less common lesion sites were the circumflex artery 8.22% (6) and smaller side branches in 6.85% (5).

During PCI, a single-stent strategy was applied to most of the vessels 87.67% (64), while two-stent and three-stent strategies were used in 10.96% (8) and 1.37% (1), respectively. All the stents used during this period were metallic second-generation drug eluting stents (*n* = 83) with a mean length size of 27.94 ± 0.41 mm and a mean diameter of 3.08 ± 0.41 mm.

### 3.3. OCT Baseline Vessel Characteristics

The baseline OCT analysis characteristics are summarized in Table 3. The median value of minimum lumen area (MLA) was 1.49 (IQR 1.12–1.93), and the minimum lumen diameter (MLD) was 1.21 (IQR 1.11–1.48). The median lesion length was 32.60 (IQR 24.05–42.30).

The presence of calcium was observed in 57.53% (42) of the vessels, and morphological characteristics were evaluated. Among all lesions, 65.12% (28) had a calcium deposit with a thickness > 0.5 mm, 53.49% (23) were more than 5 mm in length, and in 24.24% (8), the calcium arch was more than 50%.

### 3.4. Post-PCI OCT Evaluation and Optimization

Post-stenting, OCT imaging was performed in all 73 vessels to identify the appropriate stent implantation. Information regarding the SEI and MSA of the stented vessels is provided in Table 4. Additionally, qualitative and quantitative data are summarized in Table 5.

Moreover, OCT evaluation demonstrated that 35.61% (26) of the vessels were, according to our criteria, suboptimally stented, before post-PCI OCT evaluation. The percentage of vessels with stent underexpansion was 9.58% (7). This differs from the identification of 31 stented vessels with an SEI < 80%, because our criteria define stent underexpansion as a combination of SEI < 80% and MSA < 4.5 mm^2^, resulting in a smaller number of vessels meeting the underexpansion definition (Supplementary Data). Furthermore, stent malapposition was observed in 15.06% (11) of vessels. With lower occurrence, stent edge dissection was identified in 6.84% (5) of cases, and the presence of plaque burden or lipid-rich pool in the stent edges was observed in 2.73% (2) of cases. Finally, only 1.36% (1) of the cases presented tissue protrusion.

The decision for optimization was made by the interventionalist, and additional stents, along with either non-compliant or semi-compliant balloons, were used accordingly. The optimization strategy for the vessels that did not fulfill the optimal implantation criteria is summarized in Table 6.

Optimization strategy included post-dilatation for stent underexpansion with NC balloon, using a mean diameter of 3.07 ± 0.41 at 18.50 ± 4.01 atm. For stent malapposition, either NC or SC balloon was used, with mean diameters of 3.39 ± 0.57 and 3.30 ± 0.97 respectively, and a mean pressure of 17.40 ± 3.13 atm. In the case of edge dissection, an additional stent with a mean diameter of 3.20 ± 0.44 was implanted at a pressure of 15 ± 1.41 atm. Finally, in cases of plaque or lipid-rich pool at the stent edges, stents with a mean diameter of 2.37 ± 0.17 were implanted at a pressure of 15 ± 1.41 atm.

### 3.5. OCT Results (Areas, Expansion) After Stent Optimization (Included Also in Table 4)

Additional OCT imaging was undertaken after optimization to confirm the appropriate stent implantation. Of the total 30 suboptimally implanted stents, only five stented vessels (6.84%) were still not fulfilling the criteria for optimal implantation despite additional optimization maneuvers. More specifically, of the seven underexpanded stents, four (5.47%) remained with <80% expansion and MSA < 4.5 mm^2^. All previously malapposed stents were optimized successfully, similarly to all cases of edge dissection and uncovered plaque burden or lipid-rich pool. As a final note, the number of vessels with tissue protrusion remained the same at 1 (1.1%).

In terms of expansion post optimization, OCT revealed improvement in all three classifications of stent expansion indexes, with 36.99% (27) of vessels remaining with SEI < 80%, 35.61% (26) with SEI 80–90%, and 27.40% (20) achieving SEI of >90%. Additionally, improvement in the percentages of stented vessels reached MSA more than the threshold of 4.5 mm^2^ 87.67% (64), after OCT post optimization was observed as well as the median value of SEI.

## 4. Discussion

This study aimed to investigate the value of post-PCI OCT in detecting suboptimally implanted stents after angiographically appropriate stent deployment and the ability to achieve optimal results with OCT guidance. OCT findings influenced the physician’s decision, leading to additional maneuvers in 35.61% (26) of cases. Finally, 93.16% of the lesions fulfilled the currently available criteria for stent optimization.

Stent underexpansion constitutes a major predictor of stent thrombosis [10]. Results from the CLI-OPCI registry [3] identify the presence of MLA of 4.5 mm^2^ as a threshold for discriminating patients with MACE. Additionally, regarding the percentage of appropriate stent expansion, there are no uniform criteria that set a target for PCI optimization. Nevertheless, data from the DOCTORS trial showed that the cut-off value of stent expansion capable of predicting FFR > 0.90 was >79.4% [11]. Based on this information, in this study, the criterion of SEI ≥ 80% was used. We identified that 9.58% (7) stents of our sample were underexpanded, a percentage that is much lower compared to other large studies [12,13].

Moreover, in two recent studies [14,15], malapposition was a frequent finding in both acute and late stent thrombosis. OCT is an extremely reliable imaging tool for identifying stent malapposition, and in our study, we detected it in 17.4% (15) of the vessels, successfully optimizing them in all cases.

Post-PCI OCT revealed that 6.84% (5) of our sample had significant edge dissections, due to the OCT greater discriminatory capabilities. Additionally, OCT is considered to be superior in detecting even small dissections compared to other imaging modalities, such as intravascular ultrasound (IVUS) [12,16]. The criteria we used were based on results from studies that identified early stent thrombosis when cut-offs for edge dissection were defined as lateral extension > 60° and a length > 2 mm [17,18].

Optimal lesion coverage and appropriate landing zone selection in areas with plaque burden < 50% and absence of a lipid-rich pool are of great importance in the PCI strategy to avoid subsequent stent edge restenosis [17]. Although pre-PCI OCT provided important information regarding lesion morphology and plaque burden in reference segments, angiographic guidance alone at the time of stent implantation was insufficient to ensure complete lesion coverage. Accordingly, our results showed that 2.73% (2) of vessels exhibited incompletely covered lesions on post-PCI OCT, a finding consistent with previous studies [19]; additional stent implantation subsequently achieved adequate lesion coverage in all instances.

The significance of identifying tissue protrusion and the decision for further intervention are controversial. The EAPCI consensus document recognizes the presence of tissue protrusion as a finding related to poor prognosis, based on findings from two major studies, especially in cases of acute coronary syndrome (ACS) [4,17]. In contrast, IVUS studies found no adverse effect on clinical outcomes in cases of tissue protrusion [20]. This discrepancy can be clarified by the results of a multicenter registry that identified the presence of three types of tissue protrusion: smooth, disrupted fibrous tissue, and irregular protrusion. Only irregular protrusion was associated with device-oriented cardiac events. This study was designed to include only non-culprit vessels, which contributed to the rare occurrence of tissue protrusion. There was only one case (1.36%) that was considered significant > 500 μm, but no further intervention was necessary.

**Figure 3:**
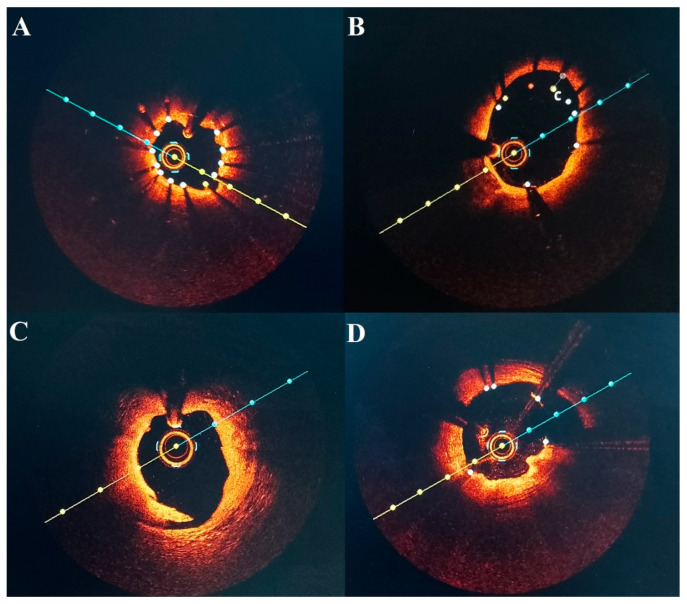
OCT imaging examples of suboptimal stent implantation. (**A**) Stent underexpansion. (**B**) Stent malapposition. (**C**) Proximal stent dissection. (**D**) Tissue protrusion through the stent struts.

We found that post-PCI OCT allowed for the identification of 30 (41.09%) suboptimal stent implantations of various types (Figure 3). Large studies that estimated incidences of suboptimal stent implantation of 31.0% (CLI-OPCI II) [8], and 35.3% (FORZA) [21] do not significantly differ from our findings. Other studies, such as (RENOVATE-COMPLEX-PCI), showed that 65.9% of those met all stent-optimization criteria [22]. In contrast, we were able to achieve a higher percentage (93.16%) of vessels that fulfilled our pre-defined criteria, results that may be influenced by the inclusion of more complex lesions, as well as the criteria for optimal stent implantation used in the RENOVATE-COMPLEX-PCI study. Furthermore, we demonstrated that even if pre–PCI OCT evaluation allowed for adequate selection of balloons and stents, significant information can be derived from post-PCI evaluation to achieve proper stent implantation criteria in most patients.

Finally, we recognize that the indications and necessity of OCT in daily practice are not clear. The European Society of Cardiology (ESC) recommends intracoronary imaging guidance by OCT during PCI on anatomically complex lesions (defined as left main coronary artery lesions, true bifurcation lesions, long lesions) (Class Ia, Level of evidence A) [23]. Several surveys by the interventional cardiologist communities showed that the most common indications for intravascular imaging were pre- and post-PCI assessment, as well as stent optimization and strategy guidance [24,25]. Our study, designed to evaluate, in everyday clinical practice, the benefits of OCT guidance, may facilitate the broader adoption of intravascular imaging by illustrating that a significant percentage of angiographically acceptable PCIs possess OCT-defined suboptimal characteristics and that post-PCI OCT-guided optimization allows operators to rectify these deficiencies, resulting in a high rate of optimal stent implantation.

## 5. Limitations

- This is a single-centered study with data collected from a limited number of patients and vessels.
- Left main lesions were not included, as OCT guidance for this subset of lesions is more cumbersome.
- Complex lesions, such as bifurcation lesions or heavily calcified lesions, were not included in this study.
- Our data may not be used to determine which types of lesions OCT may provide valuable information for stent optimization, especially due to the limited number of lesions evaluated.
- Patients with multiple stents (like additional stent implantation) may experience more procedures and a greater risk of adverse outcomes, including the need for surgery, even though in our study we did not have this situation.

## 6. Conclusions

In patients with coronary artery disease, OCT guidance after PCI provided useful information, otherwise unavailable by angiography alone. We were able to identify that 35.61% of the targeted vessels were suboptimally stented. OCT imaging was able to provide procedural and strategic guidance for optimization until the appropriate results, based on our criteria, were achieved.

## Figures and Tables

**Table 1:** Baseline characteristics of the sample.

	All Patients (*n* = 64)
Age, y	63.03 ± 0.40
Male sex	79.69% (51)
Diabetes mellitus	21.87% (14)
Hypertension	84.37% (54)
Dyslipidemia	87.50% (56)
Ever smoker	65.63% (42)
History of angina	53.97% (34)
History of ACS	29.69% (19)
LVEF,%	48.10 ± 6.32
eGFR, mL/min/1.73 m^2^	89.93 ± 17.25
Clinical presentation	
STEMI	53.12% (34)
NSTEMI	6.25% (4)
UA	37.50% (24)
Stable angina	3.13% (2)
Number of vessels diseased	
Single vessel disease	26.56% (17)
Double vessel disease	60.94% (39)
Triple vessel disease	12.50% (8)
Mean number of vessels	1.85 ± 0.63

ACS, acute coronary syndrome; LVEF, left ventricular ejection fraction; eGFR, estimated glomerular filtration rate; STEMI, ST-elevation myocardial infarction; NSTEMI, non ST-elevation myocardial infarction; UA, unstable angina.

**Table 2:** Lesion and treatment characteristics.

	Total Vessels (*n* = 73)
Lesion location	
Left anterior descending artery	64.38% (47)
Right coronary artery	20.55% (15)
Circumflex artery	8.22% (6)
Side branches	6.85% (5)
Number of stents implanted	
1	87.67% (64)
2	10.96% (8)
3	1.37% (1)
Mean number of stents	1.13 ± 0.38
Stent characteristics	
Metallic 2nd-generation drug eluting stent	100%
Stent length, mm	27.94 ± 0.41
Stent diameter, mm^2^	3.08 ± 0.41

**Table 3:** Baseline vessel characteristics using optical coherence tomography.

	Total Vessels (*n* = 73)
Lumen measurements	
Proximal lumen area, mm^2^	7.89 (6.41–10.65)
Proximal lumen diameter, mm	3.17 (2.83–3.61)
Distal lumen area, mm^2^	5.53 (4.13–7.38)
Distal lumen diameter, mm	2.70 (2.32–3.06)
Average reference lumen area, mm^2^	6.86 (5.76–8.84)
Average reference lumen diameter, mm	2.93 (2.63–3.27)
Minimum lumen area, mm^2^	1.49 (1.12–1.93)
Minimum lumen diameter, mm	1.21 (1.11–1.48)
Lesion length, mm	32.60 (24.05–42.30)
Calcium characteristics	
Presence of calcium	57.53% (42)
Thickness > 0.5 mm	65.12% (28)
Angle > 180° vessel arc	24.24% (8)
Length > 5 mm	53.49% (23)

Data shown as median (interquartile range) or percentages.

**Table 4:** OCT post-PCI stent expansion characteristics.

	OCT Post-PCI (*n* = 73)	OCT Post Optimization (*n* = 73)
Stent expansion index classification, %		
<80%	42.46% (31)	36.99% (27)
80–90%	31.51% (23)	35.61% (26)
>90%	26.03% (19)	27.40% (20)
Stent measurements		
Minimum stent area > 4.5 mm^2^	82.19% (60)	87.67% (64)
Minimum stent area < 4.5 mm^2^	17.81% (13)	12.33% (9)
Minimum stent area, mm^2^	5.44 (4.61–7.16)	5.46 (4.72–7.32)
Stent expansion index	81.15 (71.42–90.48)	81.51 (73.50–91.00)

Data shown as median (interquartile range) or percentages.

**Table 5:** OCT identification of suboptimal stent implantation before and after optimization.

	OCT Post-PCI (*n* = 73)	OCT Post Optimization (*n* = 73)
Suboptimal stent implantation measurements		
Total suboptimally stented vessels	35.61% (26)	6.84% (5)
Stent underexpansion	9.58% (7)	5.47% (4)
Stent malapposition	15.06% (11)	0.0% (0)
Edge dissection	6.84% (5)	0.0% (0)
Plaque burden or lipid-rich pool	2.73% (2)	0.0% (0)
Tissue protrusion (plaque/thrombus)	1.36% (1)	1.36% (1)

**Table 6:** Optimization strategy equipment.

	NC Balloon Diameter, mm	SC Balloon Diameter, mm	Stent Diameter, mm	Pressure, atm
Suboptimal stent implantation				
Stent underexpansion	3.07 ± 0.41	-	-	18.50 ± 4.01
Stent malapposition	3.39 ± 0.57	3.30 ± 0.97	-	17.40 ± 3.13
Edge dissection	-	-	3.20 ± 0.44	15 ± 1.41
Plaque burden or lipid rich pool	-	-	2.37 ± 0.17	15 ± 1.41

NC balloon, non-compliant balloon; SC balloon, semi-compliant balloon; ATM, atmosphere.

## Data Availability

The data supporting the conclusions of this article will be made available by the authors upon request.

## Supplementary Materials

The following supporting information can be downloaded at: https://www.mdpi.com/article/10.3390/diagnostics16050813/s1, Supplementary Data: Stent expansion indexes and MSA of all vessels in the study post-PCI and post-optimization.

## References

[1] Prati F., Di Vito L., Biondi-Zoccai G., Occhipinti M., La Manna A., Tamburino C., Burzotta F., Trani C., Porto I., Ramazzotti V. (2012). Angiography alone versus angiography plus optical coherence tomography to guide decision-making during percutaneous coronary intervention: The Centro per la Lotta contro l’Infarto-Optimisation of Percutaneous Coronary Intervention (CLI-OPCI) study. EuroIntervention.

[2] Vergallo R., Ren X., Yonetsu T., Kato K., Uemura S., Yu B., Jia H., Abtahian F., Aguirre A.D., Tian J. (2014). Pancoronary plaque vulnerability in patients with acute coronary syndrome and ruptured culprit plaque: A 3-vessel optical coherence tomography study. Am. Heart J..

[3] Prati F., Romagnoli E., La Manna A., Burzotta F., Gatto L., Marco V., Fineschi M., Fabbiocchi F., Versaci F., Trani C. (2018). Long-term consequences of optical coherence tomography findings during percutaneous coronary intervention: The Centro Per La Lotta Contro L’infarto—Optimization Of Percutaneous Coronary Intervention (CLI-OPCI) LATE study. EuroIntervention.

[4] Sreenivasan J., Reddy R.K., Jamil Y., Malik A., Chamie D., Howard J.P., Nanna M.G., Mintz G.S., Maehara A., Ali Z.A. (2024). Intravascular Imaging-Guided Versus Angiography-Guided Percutaneous Coronary Intervention: A Systematic Review and Meta-Analysis of Randomized Trials. J. Am. Heart Assoc..

[5] Truesdell A.G., Alasnag M.A., Kaul P., Rab S.T., Riley R.F., Young M.N., Batchelor W.B., Maehara A., Welt F.G., Kirtane A.J. (2023). Intravascular Imaging During Percutaneous Coronary Intervention: JACC State-of-the-Art Review. J. Am. Coll. Cardiol..

[6] Ali Z.A., Shin D., Chaturvedi A., Waksman R. (2024). We now have enough evidence to support systematic OCT in daily PCI practice: Pros and cons. EuroIntervention.

[7] Ploscaru V., Popa-Fotea N.M., Calmac L., Itu L.M., Mihai C., Bataila V., Dragoescu B., Puiu A., Cojocaru C., Costin M.A. (2022). Artificial intelligence and cloud based platform for fully automated PCI guidance from coronary angiography-study protocol. PLoS ONE.

[8] Räber L., Mintz G.S., Koskinas K.C., Johnson T.W., Holm N.R., Onuma Y., Radu M.D., Joner M., Yu B., Jia H. (2018). Clinical use of intracoronary imaging. Part 1: Guidance and optimization of coronary interventions. An expert consensus document of the European Association of Percutaneous Cardiovascular Interventions. Eur. Heart J..

[9] Prati F., Romagnoli E., Burzotta F., Limbruno U., Gatto L., La Manna A., Versaci F., Marco V., Di Vito L., Imola F. (2015). Clinical Impact of OCT Findings During PCI: The CLI-OPCI II Study. JACC Cardiovasc. Imaging.

[10] Fujii K., Carlier S.G., Mintz G.S., Yang Y.M., Moussa I., Weisz G., Dangas G., Mehran R., Lansky A.J., Kreps E.M. (2005). Stent underexpansion and residual reference segment stenosis are related to stent thrombosis after sirolimus-eluting stent implantation: An intravascular ultrasound study. J. Am. Coll. Cardiol..

[11] Meneveau N., Souteyrand G., Motreff P., Caussin C., Amabile N., Ohlmann P., Morel O., Lefrançois Y., Descotes-Genon V., Silvain J. (2016). Optical Coherence Tomography to Optimize Results of Percutaneous Coronary Intervention in Patients with Non-ST-Elevation Acute Coronary Syndrome: Results of the Multicenter, Randomized DOCTORS Study (Does Optical Coherence Tomography Optimize Results of Stenting). Circulation.

[12] Wijns W., Shite J., Jones M.R., Lee S.W.L., Price M.J., Fabbiocchi F., Barbato E., Akasaka T., Bezerra H., Holmes D. (2015). Optical coherence tomography imaging during percutaneous coronary intervention impacts physician decision-making: ILUMIEN I study. Eur. Heart J..

[13] Soeda T., Uemura S., Park S.J., Kim J.S., Lee S.J., Itoh T., Yonetsu T., Kakuta T., Jang I.K. (2015). Incidence and Clinical Significance of Poststent Optical Coherence Tomography Findings: One-Year Follow-Up Study from a Multicenter Registry. Circulation.

[14] Adriaenssens T., Joner M., Godschalk T.C., Malik N., Alfonso F., Xhepa E., De Cock D., Komukai K., Tada T., Cuesta J. (2017). Optical Coherence Tomography Findings in Patients with Coronary Stent Thrombosis: A Report of the PRESTIGE Consortium (Prevention of Late Stent Thrombosis by an Interdisciplinary Global European Effort). Circulation.

[15] Souteyrand G., Amabile N., Mangin L., Chabin X., Meneveau N., Cayla G., Vanzetto G., Barnay P., Trouillet C., Rioufol G. (2016). Mechanisms of stent thrombosis analysed by optical coherence tomography: Insights from the national PESTO French registry. Eur. Heart J..

[16] Ali Z.A., Maehara A., Généreux P., Shlofmitz R.A., Fabbiocchi F., Nazif T.M., Guagliumi G., Meraj P.M., Alfonso F., Samady H. (2016). Optical coherence tomography compared with intravascular ultrasound and with angiography to guide coronary stent implantation (ILUMIEN III: OPTIMIZE PCI): A randomised controlled trial. Lancet.

[17] Choi S.Y., Witzenbichler B., Maehara A., Lansky A.J., Guagliumi G., Brodie B., Kellett M.A., Dressler O., Parise H., Mehran R. (2011). Intravascular ultrasound findings of early stent thrombosis after primary percutaneous intervention in acute myocardial infarction: A Harmonizing Outcomes with Revascularization and Stents in Acute Myocardial Infarction (HORIZONS-AMI) substudy. Circ. Cardiovasc. Interv..

[18] Cheneau E., Leborgne L., Mintz G.S., Kotani J.I., Pichard A.D., Satler L.F., Canos D., Castagna M., Weissman N.J., Waksman R. (2003). Predictors of subacute stent thrombosis: Results of a systematic intravascular ultrasound study. Circulation.

[19] Rai H., Harzer F., Otsuka T., Abdelwahed Y.S., Antuña P., Blachutzik F., Koppara T., Räber L., Leistner D.M., Alfonso F. (2022). Stent Optimization Using Optical Coherence Tomography and Its Prognostic Implications After Percutaneous Coronary Intervention. J. Am. Heart Assoc..

[20] Qiu F., Mintz G.S., Witzenbichler B., Metzger D.C., Rinaldi M.J., Duffy P.L., Weisz G., Stuckey T.D., Brodie B.R., Parvataneni R. (2016). Prevalence and Clinical Impact of Tissue Protrusion After Stent Implantation: An ADAPT-DES Intravascular Ultrasound Substudy. JACC Cardiovasc. Interv..

[21] Burzotta F., Leone A.M., Aurigemma C., Zambrano A., Zimbardo G., Arioti M., Vergallo R., De Maria G.L., Cerracchio E., Romagnoli E. (2020). Fractional Flow Reserve or Optical Coherence Tomography to Guide Management of Angiographically Intermediate Coronary Stenosis: A Single-Center Trial. JACC Cardiovasc. Interv..

[22] Lee J.M., Choi K.H., Song Y.B., Lee J.Y., Lee S.J., Lee S.Y., Kim S.M., Yun K.H., Cho J.Y., Kim C.J. (2023). Intravascular Imaging-Guided or Angiography-Guided Complex PCI. N. Engl. J. Med..

[23] Vrints C., Andreotti F., Koskinas K.C., Rossello X., Adamo M., Ainslie J., Banning A.P., Budaj A., Buechel R.R., Chiariello G.A. (2024). 2024 ESC Guidelines for the management of chronic coronary syndromes. Eur. Heart J..

[24] Koskinas K.C., Nakamura M., Räber L., Colleran R., Kadota K., Capodanno D., Wijns W., Akasaka T., Valgimigli M., Guagliumi G. (2018). Current use of intracoronary imaging in interventional practice—Results of a European Association of Percutaneous Cardiovascular Interventions (EAPCI) and Japanese Association of Cardiovascular Interventions and Therapeutics (CVIT) Clinical Practice Survey. EuroIntervention.

[25] Low A.F., Wongpraparut N., Chunhamaneewat N., Jeamanukoolkit A., Jhung L.T., Zhen-Vin L., Tan C.T., Hwa H.H., Rajagopal R., Yahya A.F. (2023). Clinical use of optical coherence tomography during percutaneous coronary intervention and coronary procedures in Southeast Asia: A survey-based expert consensus summary. AsiaIntervention.

