# Supplementary material for: Assessment of Optimal Stent Implantation with the Use of Optical Coherence Tomography in Patients with Coronary Artery Disease
Source: Diagnostics (Basel). 2026 Mar 9;16(5):813. doi: 10.3390/diagnostics16050813 (PMC12984583; doi:10.3390/diagnostics16050813)
Supplement: Supplementary file 1 [file diagnostics-16-00813-s001.zip › diagnostics-4033042-supplementary.pdf]

SUPPLEMENTARY DATA

Table: Stent expansion indexes and MSA of all vessels in the study post-PCI and post-optimization

NO. OF VESSEL	POST PCI SEI	POST PCI-MSA	POST-OPT SEI	POST-OPT MSA
1	97,64	7,05	97,65	7,05
2	70,79	6,06	70,79	6,06
3	82,61	8,1	82,61	8,1
4	81,15	6,72	81,16	6,72
5	61,69	3,56	67,00	3,89
6	104,28	9,5	104,00	9,5
7	87,20	4,6	87,00	4,6
8	87,53	5,3	87,53	5,3
9	88,87	11,66	88,87	11,66
10	74,63	8,15	74,63	8,15
11	86,17	8,6	86,00	8,6
12	68,36	4,7	68,36	4,7
13	91,31	8,2	91,31	8,2
14	81,33	9,96	81,34	9,96
15	61,38	5,38	68,00	5,96
16	80,05	5,42	80,06	5,42
17	72,06	5,61	72,06	5,61
18	89,73	7,08	89,73	7,08
19	90,54	3,88	90,55	3,88
20	85,69	5,87	85,69	5,87
21	77,50	5,1	77,51	5,1
22	90,42	4,63	90,43	4,63
23	75,96	3,43	75,97	3,43
24	81,51	6,02	81,52	6,02
25	92,03	5,55	92,04	5,55
26	106,69	7,81	106,69	7,81
27	117,42	6,03	117,43	6,03
28	92,71	9,8	92,72	9,8
29	100	11,16	100,00	11,16
30	76,08	5,44	76,08	5,44
31	92,34	7,72	92,34	7,72
32	91,01	7,04	91,01	7,04
33	66,17	4,5	66,18	4,5
34	67,74	4,39	73,00	4,79
35	88,14	5,24	88,14	5,24
36	60,02	5,18	60,02	5,18
37	83,08	6,31	83,08	6,31
38	80	5,42	80,00	5,42
39	93,01	4,26	93,01	4,26
40	76,13	5,44	78,00	5,55
41	43,99	4,85	47,00	5,21
42	73,06	5,71	91,00	7,17
43	74,42	9,75	74,00	9,75
44	66,35	4,97	66,36	4,97
45	49,34	4,51	49,34	4,51
46	55,14	2,68	64,00	3,09

47	112,97	9,23	112,97	9,23
48	97,50	3,91	97,51	3,91
49	60,22	5,11	60,22	5,11
50	65,43	8,69	65,44	8,69
51	83,76	4,51	83,77	4,51
52	80,38	3,3	80,39	3,3
53	78,49	7,81	78,49	7,81
54	59,32	3,77	59,32	3,8
55	95,85	4,74	95,85	4,74
56	86,57	5,77	86,57	5,77
57	88,85	3,35	88,86	3,35
58	66,75	5,3	67,75	5,4
59	78,66	4,94	78,66	4,94
60	103,33	2,94	103,34	2,94
61	98,04	10,01	98,04	10,01
62	73,96	6,32	86,00	7,38
63	86,67	4,91	86,67	4,91
64	80,37	7,27	80,38	7,27
65	86,44	7,24	86,45	7,24
66	17,96	1,61	53,00	4,8
67	79,28	6,43	92,00	7,53
68	60,87	4,38	80,00	5,8
69	80,75	6,23	80,75	6,23
70	93,50	5,4	93,51	5,4
71	80,06	4,64	80,07	4,64
72	74,81	4,53	74,81	4,53
73	66,34	5,46	66,34	5,46
74	80,14	5,97	81,89	6,11

PCI, Percutaneous coronary intervention; SEI, Stent Expansion Index; OPT, optimization.
